# Supplementary material for: Evaluating the content and face validity of Arabic-translated Patient Measures of Safety survey PMOS-30
Source: PLoS One. 2024 Jun 13;19(6):e0304151. doi: 10.1371/journal.pone.0304151 (PMC11175530; doi:10.1371/journal.pone.0304151)
Supplement: Attachment — Submitted filename: plosone.docx [file pone.0304151.s002.docx]

1) All of psychometric characteristics of tool should be examined.
2) The author has mentioned tools for measurement safety patient and satisfaction, it is necessary to mention these tools in the introduction.
3) The authors need to provide a more in-depth discussion about what the constructs in this measure are supposed to be measuring.
4) I would also encourage the authors to again pay close attention to grammar throughout the manuscript
5) The authors need to provide a more in-depth discussion about what the constructs in this measure are supposed to be measuring.
6) The discussion needs more discussion part.
7) Tool structures need more discussion and analysis.
