# Supplementary material for: Evaluating the content and face validity of Arabic-translated Patient Measures of Safety survey PMOS-30
Source: PLoS One. 2024 Jun 13;19(6):e0304151. doi: 10.1371/journal.pone.0304151 (PMC11175530; doi:10.1371/journal.pone.0304151)
Supplement: Attachment — Submitted filename: Response to Reviewers Plosone 20-10-2023.docx [file pone.0304151.s003.docx]

**Evaluating the content and face validity of Arabic-translated Patient Measures of Safety Survey PMOS-30**

Dear All,

Thank you very much for your valuable comments. We really appreciate your effort and time spent on our manuscripts. Please find below the comment and our response to the new line.

Best Regards,

Rabab

| **Comments** | **Response** | **Line** |
| --- | --- | --- |
| Reviewer #1: |  |  |
| 1) All of psychometric characteristics of tool should be examined | Thank you very much for your comments.  Agree it is ideal to test all the psychometric properties it is acceptable in the literature to test partially, like the validity and acceptability.  Also, the psychometric properties were tested in the below reference.   G, Reynolds C, Moore S, et al. Validation of revised patient measures of safety: PMOS-30 and PMOS-10. BMJ Open 2019;9:e031355. doi:10.1136/ bmjopen-2019-031355 |  |
| 2) The author has mentioned tools for measurement safety patient and satisfaction, it is necessary to mention these tools in the introduction. | Agree, and added. | 55- 75 |
| 3) The authors need to provide a more in-depth discussion about what the constructs in this measure are supposed to be measuring. | Agree, and added. | 271-281 |
| 4) I would also encourage the authors to again pay close attention to grammar throughout the manuscript. | Agree, and added. | All over the script |
| 5) The authors need to provide a more in-depth discussion about what the constructs in this measure are supposed to be measuring | Considered in the point 3 |  |
| 6) The discussion section needs a deeper expansion, it is necessary to compare and discuss all the results with similar studies. | Agree, and added. | 262, 267, 296-319 |
| 7) Please provide additional details regarding participant consent. In the ethics statement in the Methods and online submission information, please ensure that you have specified what type you obtained (for instance, written or verbal, and if verbal, how it was documented and witnessed). If your study included minors, state whether you obtained consent from parents or guardians. If the need for consent was waived by the ethics committee, please include this information. | Added  Participants were fully informed via a link about the study aims and the use of their data, and their consent was provided verbally before participating in the study. | 200, 201 |
| Reviewer #2: |  |  |
| Thank you for conducting this interesting study. I have concerns about the followings: 1. There is lack of clarity about content validity in terms of: • I do not think you have followed Lynns’ Method for content validity? Please follow and refer (Lynn, M.R. (1986) Determination and quantification of content validity. Nursing Research, 35 (6), 382- 385.) • The underlying context • The different levels od accepting or deleting each item • The description of the experts who participated | Thank you very much for your valuable comment.  We have not used the Lynn method, however, this tool have been test for it content validity when it was developed. Since this is copyrighted, we get permission to translate and follow the scientific methodology to validate the translation.  We have used 2 methods and we repeated them after the amendments   1- Patients 2- Raters   As we are not permitted to delete items, items who has issues from the second interview were returned to the expert to solve them   - Added the Expert Description | 144- 148 |
| 2. The number of experts is minimum to be accepted | Yes however it has been used in the literature | 297 |
| 3. The threshold for keeping any item is 0.78 not 0.75 | Agree and corrected |  |
| 4. Keeping item when it received less than 0.78 I-CVI is not recommended. How have you restructure them to be kept in the questionnaire? | As we mentioned before, we cannot delete any item for the copyright. We revisit the questions and compare them with patients' results in the table. If there was disagreement, we made the changes. | 236-237 |
| 5. The term test-retest is confusing with test-retest reliability so I prefer to use “second round of validation” instead | Thank you for your comment, changed accordingly | 171, 174, 260 |
| 6. I suggest testing the internal consistency reliability with Cronbach’s alpha test | It is a good suggestion; however, there will be many tests in this paper which could confuse the readers. |  |

.
